# Engineering of mCherry variants with long Stokes shift, red-shifted fluorescence, and low cytotoxicity

**DOI:** 10.1371/journal.pone.0171257

**Published:** 2017-02-27

**Authors:** Yi Shen, Yingche Chen, Jiahui Wu, Nathan C. Shaner, Robert E. Campbell

**Affiliations:** 1 Department of Chemistry, University of Alberta, Edmonton, Alberta, Canada; 2 Department of Photobiology and Bioimaging, The Scintillon Institute, San Diego, California, United States of America; The Francis Crick Institute, UNITED KINGDOM

## Abstract

MCherry, the *Discosoma* sp. mushroom coral-derived monomeric red fluorescent protein (RFP), is a commonly used genetically encoded fluorophore for live cell fluorescence imaging. We have used a combination of protein design and directed evolution to develop mCherry variants with low cytotoxicity to *Escherichia coli* and altered excitation and emission profiles. These efforts ultimately led to a long Stokes shift (LSS)-mCherry variant (λ_ex_ = 460 nm and λ_em_ = 610 nm) and a red-shifted (RDS)-mCherry variant (λ_ex_ = 600 nm and λ_em_ = 630 nm). These new RFPs provide insight into the influence of the chromophore environment on mCherry’s fluorescence properties, and may serve as templates for the future development of fluorescent probes for live cell imaging.

## Introduction

The discovery of red fluorescent proteins (RFPs) in non-bioluminescent Anthozoa species [1] was a breakthrough that rivals the inimitable discovery of *Aequorea victoria* green fluorescent protein (avGFP) [2,3], and the first examples of recombinant expression of avGFP and its color variants [4–7]. Subsequent protein engineering efforts have produced two predominant lineages of monomeric RFPs derived from naturally tetrameric precursors. One lineage is derived from *Discosoma* sp. mushroom coral and includes the first monomeric (m) RFP, mRFP1 [8], and the mRFP1-derived “mFruit” variants mCherry, mOrange [9], and mApple [10], among others. mCherry is currently the most widely used mRFP for live cell imaging due to its monomeric structure, high brightness, fast maturation, and good photostability [9]. The second lineage is engineered from the *Entacmaea quadricolor* sea anemone RFPs eqFP578 and eqFP611 [11] and includes TagRFP [12], mKate [13], mKate2 [14], mRuby [15], mRuby2 [16], mRuby3 [17], and FusionRed [18]. All of the above mentioned RFPs have been widely used by the cell biology research community for imaging of protein dynamics and localization in live cells.

A substantial effort has been invested in modifying the properties of typical RFPs (that is, those with λ_ex_ ~ 570–590 nm and λ_em_ ~ 590–620 nm) in order to create variants that exhibit either long Stokes shift fluorescence (λ_ex_ ~ 440–460 nm and λ_em_ ~ 590–620 nm, Stokes shift ≥ 100 nm) or red-shifted fluorescence (λ_ex_ ≥ 600 nm and λ_em_ ≥ 630 nm). Red-shifted RFPs are desirable for *in vivo* imaging applications because longer wavelength light is associated with decreased tissue scattering, absorbance, and autofluorescence [19]. Long Stokes shift RFPs are particularly useful for use with two-photon laser-scanning fluorescence imaging because their maximal two-photon excitation wavelengths are similar to those of enhanced avGFP (EGFP) and well within the range of typical Ti-Sapphire lasers [20].

Long Stokes shift fluorescence of fluorescent proteins occurs when the p*K*_a_ of the neutral phenol form of the chromophore substantially decreases upon excitation, leading to excited state proton transfer (ESPT) and emission of fluorescence from the anionic phenolate form [21]. As the phenol form is higher energy than the phenolate form, the energy difference between excitation and emission (i.e., the Stokes shift) is increased relative to typical fluorescent proteins that do not undergo ESPT. The first reported long Stokes shift RFP was a variant known as mKeima that was engineered from a chromoprotein from the stony coral *Montipora* sp. [22]. Later examples included LSSmKate1, LSSmKate2 [23], and mBeRFP [24], which were engineered from mKate through the rational introduction of an ESPT pathway. An analogous strategy has been used to introduce an ESPT pathway into the mFruit RFPs mOrange and mCherry [25]. However, the resulting long Stokes shift mCherry variants contained a mixture of both green and red emitting species.

Relative to the mechanism that leads to long Stokes shift RFP fluorescence (i.e., ESPT), the mechanisms that lead to red-shifted RFP fluorescence are not as well understood. In principle, red-shifted fluorescence must result from a decrease in the energy difference between the ground state and the excited state of the chromophore, as dictated by the interactions of the chromophore with its proteinaceous environment. For example, in mPlum (λ_ex_ = 590 nm and λ_em_ = 649 nm) [26], one of the only variants that has been investigated in detail, the red-shifted fluorescence is attributed to a dynamic Stokes shift resulting from an interaction between the chromophore and a conformationally dynamic glutamate side chain [27]. Empirically-driven directed evolution efforts have produced a number of additional red-shifted RFPs including the mRFP1-derived mRaspberry (λ_ex_ = 598 nm and λ_em_ = 625 nm) [26], mGrape3 (λ_ex_ = 608 nm and λ_em_ = 646 nm) [28], and mRouge (λ_ex_ = 600 nm and λ_em_ = 637 nm) [29]. Similarly, red-shifted variants of the sea anemone-derived mKate RFP, including mCardinal (λ_ex_ = 604 nm and λ_em_ = 659 nm) [30] and TagRFP657 (λ_ex_ = 611 nm and λ_em_ = 657 nm) [31], have been developed.

In this report we describe efforts to engineer new long Stokes shift and red-shifted RFPs based on mCherry variants with decreased cytotoxicity. These new RFP variants provide insight into the amino acid determinants of mCherry fluorescence color, and may serve as templates for further expansion of the series of mFruit fluorophores [9,10] and mFruit-derived reporters [32–34].

## Results

### mCherry2 as template for directed evolution

**Fig 1:**
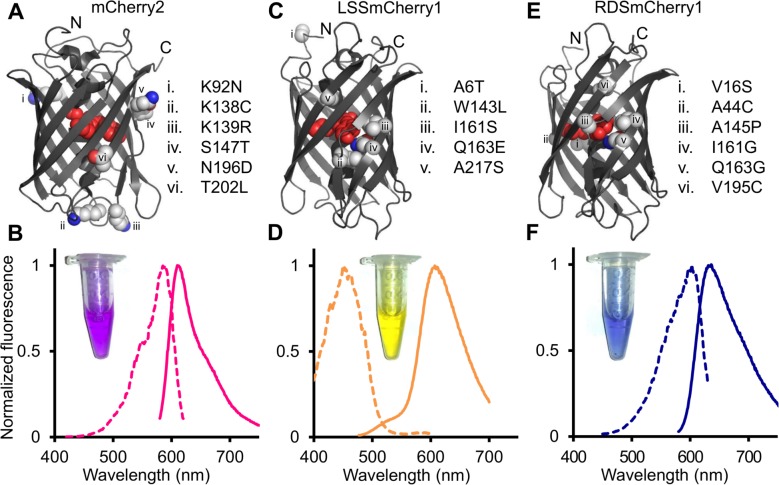
Summary of mutations and fluorescence spectra of mCherry2, LSSmCherry1, and RDSmCherry1. (A,C,E) Positions of amino acid substitutions mapped onto the crystal structure of mCherry (PDB ID: 2H5Q) [50] for (A) mCherry2, (B) LSSmCherry1, and (C) RDSmCherry1. (B,D,F) Normalized fluorescence excitation (dashed line) and emission spectra (solid line) of (B) mCherry2, (D) LSSmCherry1, and (F) RDSmCherry1. Insets are white light images of purified proteins.

**Fig 2:**
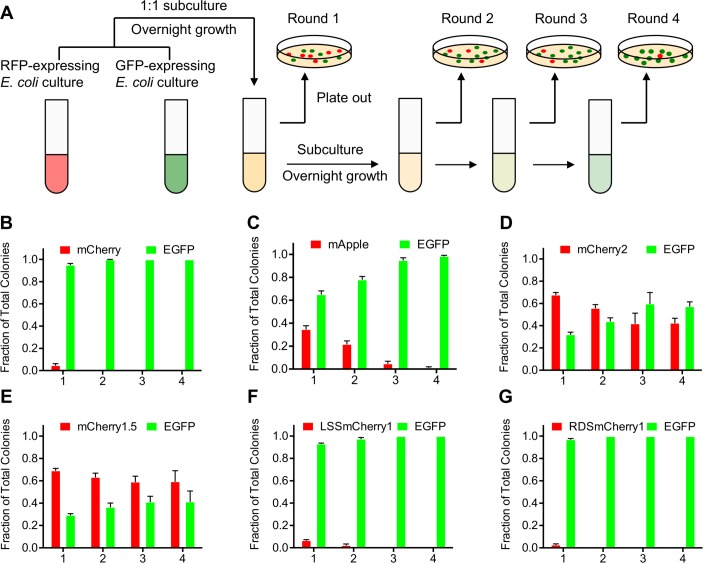
Bacterial cytotoxicity assay. (A) Schematic representation of the workflow used to evaluate the cytotoxicity of RFPs. (B-G) Results obtained when the assay represented in (A) was applied to (B) mCherry, (C) mApple, (D) mCherry2, (E) mCherry1.5, (F) LSSmCherry1, and (G) RDSmCherry1 (n = 3 for each FP variant). For each chart, numbers along the x-axis refer to the round of subculturing as indicated in (A). Error bars represent standard deviation.

The mFruit variant mCherry2 (mCherry-K92N/K138C/K139R/S147T/N196D/T202L) is a further engineered variant of mCherry that retains similar excitation and emission maxima (λ_ex_ = 589 nm and λ_em_ = 610 nm) but has slightly higher brightness (Fig 1A and 1B; Table 1). This variant was generated some years ago by several rounds of directed evolution of an mCherry-ferritin fusion, as was originally described for Superfolder GFP [35], but was not characterized in detail until the current work. The increased *in vitro* brightness of mCherry2 does not translate into substantially improved brightness in either bacteria or mammalian cells. However, we noticed that *E*. *coli* colonies expressing mCherry2 have a larger size than the ones expressing mCherry, at similar colony densities on LB agar plates. This observation suggested that mCherry2 might have decreased cytotoxicity in *E*. *coli*. To further explore this possibility, a bacterial growth rate comparison test to evaluate the cytotoxicity of RFPs, relative to an EGFP standard, was developed (Fig 2A). *E*. *coli* bearing RFP or EGFP encoding plasmids with the same promoter were first cultured overnight separately without induction of protein expression. The following day, 50 μL from both *E*. *coli* cultures were mixed in a 1:1 (v/v) ratio, and then diluted into 4 mL of fresh medium with inducer. Dilution was repeated every day for 4 days and a small portion of each overnight culture was plated on agar plates supplemented with inducer. Green and red fluorescence images of the plates were acquired, and percentages of EGFP and RFP-expressing bacteria were calculated based on counting of the number of fluorescent colonies in each image. Using this assay, the relative cytotoxicity of mCherry [9], mApple [10], and mCherry2 was evaluated relative to EGFP (Fig 2B–2D). These tests revealed that the population of bacteria expressing EGFP quickly overtook the population of bacteria expressing mCherry and mApple (Fig 2B and 2C). We interpret this result as indicating that bacteria expressing mCherry or mApple had a slower growth rate due to higher cytotoxicity of mCherry relative to EGFP. In contrast, bacteria expressing mCherry2 (Fig 2D) showed similar growth rates with bacteria expressing EGFP in the time frame of this test, suggesting they had lower cytotoxicity than mCherry. Due to the high brightness and low cytotoxicity, mCherry2 was chosen as the template for subsequent development of long Stokes shift and red-shifted variants.

**Table 1:** Spectral properties of RFPs. Substitutions for all new variants are provided in S1 Table.

	Fluorescent Protein Name	Excitation Maximum (nm)	Emission Maximum (nm)	ε (M^−1^ cm^−1^)	Φ	Brightness (ε * Φ / 1000)	p*K*_a_
	mCherry [9]	587	610	72,000	0.22	15.8	<4.5
	mCherry2	589	610	79,400 ± 300	0.22 ± 0.01	17.5 ± 0.7	3.3 ± 0.2
Long Stokes shift	LSSmCherry1	450	610	35,000 ± 500	0.29 ± 0.04	10.1 ± 0.9	6.2 ± 0.1
	LSSmKate1 [23]	463	624	31,200	0.08	2.5	3.2
	LSSmKate2 [23]	460	605	26,000	0.17	4.4	2.7
	mKeima [22]	440	620	14,400	0.24	3.5	6.5
	mBeRFP [24]	446	611	65,000	0.27	17.5	5.6
	hmKeima8.5 [44]	438	612	32,000	0.34	10.9	5.3
	CyOFP1 [45]	497, 523	589	40,000	0.76	30.4	5.5
Red-shifted	RDSmCherry0.1	598	625	59,600 ± 100	0.10 ± 0.02	6.0 ± 0.9	N.D.
	RDSmCherry0.2	600	630	34,400 ± 200	0.03 ± 0.01	1.0 ± 0.2	N.D.
	RDSmCherry0.5	604	636	23,300 ± 300	0.02 ± 0.01	0.5 ± 0.2	N.D.
	RDSmCherry1	600	630	55,400 ± 200	0.09 ± 0.01	5.0 ± 0.6	5.6 ± 0.1
	mRouge [29]	600	637	43,000	0.02	0.9	6.1
	TagRFP657 [31]	611	657	34,000	0.10	3.4	3.4
	mCardinal [30]	604	659	87,000	0.19	16.5	5.3

(n = 3, for extinction coefficient, quantum yield, brightness, and p*K*_a_ measurements of all new variants).

In an effort to identify the mutations responsible for the decreased cytotoxicity of mCherry2, we generated a number of variants with various combinations of mCherry2 mutations using site directed mutagenesis. The mCherry-K92N/K138C/K139R/N196D variant, with just four of the six mCherry2 mutations, permitted a relative faster *E*. *coli* growth rate than EGFP (Fig 2E), suggesting that this variant (designated as mCherry1.5) had even lower cytotoxicity than EGFP and mCherry2. To investigate whether the low bacterial toxicity was correlated with improved performance for mammalian cell imaging (possibly due to a decreased tendency to non-specifically aggregate [36]), mCherry1.5 was expressed in mammalian cells as a fusion to the cytoplasmic end of an endoplasmic reticulum signal-anchor membrane protein (CytERM) [37] and calcium release-activated calcium channel protein 1 (Orai1), which both tend to mislocalize when fused to mCherry [38]. Unfortunately, both mCherry1.5 and mCherry itself showed similar patterns of protein mislocalization in these fusions, as compared to identical mEGFP fusions (S1 Fig), indicating that the low bacterial cytotoxicity of an RFP does not necessarily correlate with more faithful fusion protein localization. Based on this result and our cumulative experience with engineering RFPs, we suggest that ameliorating the mislocalization of RFPs may ultimately require a high-throughput image-based screen of mammalian cells expressing a library of RFP variants in the context of a fusion that tends to mislocalize.

### Directed evolution of a long Stokes shift mCherry variant

**Fig 3:**
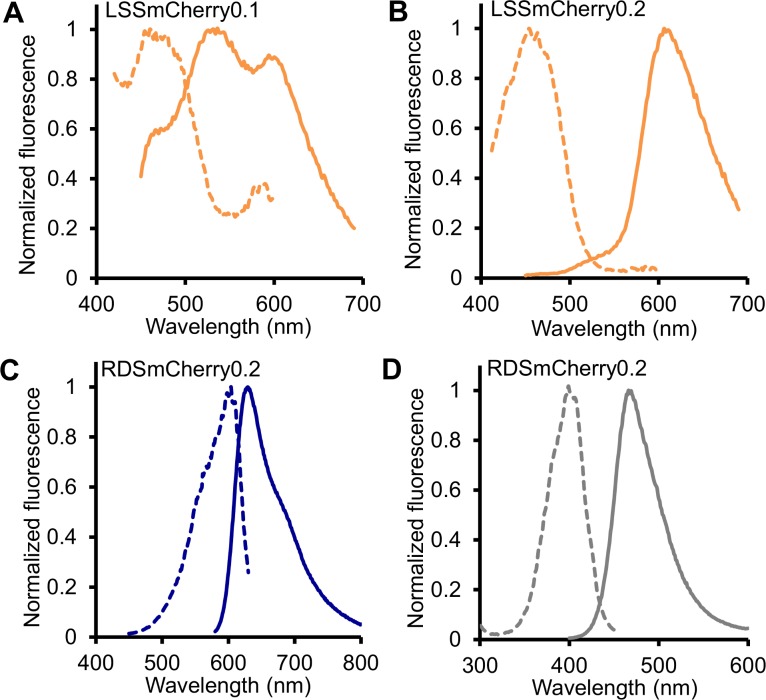
Fluorescence profiles of key intermediates in the directed evolution of LSSmCherry and RDSmCherry. Normalized fluorescence excitation (dashed line) and emission spectra (solid line) of (A) LSSmCherry0.1, (B) LSSmCherry0.2, (C) the red fluorescent component of RDSmCherry0.2, and (D) the blue fluorescent component of RDSmCherry0.2.

To engineer a long Stokes shift mCherry variant, a combination of semi-rational design and random mutagenesis were performed, starting from the template of the mCherry2 gene. In the first step, site-directed saturation mutagenesis was performed at residues 161 and 163 of mCherry2 (numbered according to DsRed) in an effort to introduce an excited state proton transfer (ESPT) pathway [25]. The library of bacterial clones expressing mCherry variants was screened in the context of colonies using a custom-built fluorescent colony screening system [39]. This screen was aimed at identification of colonies that exhibited maximal long Stokes shift red fluorescence (λ_ex_ = 470/40 nm and λ_em_ = 630/60 nm) and minimal short Stokes shift fluorescence (λ_ex_ = 560/40 nm and λ_em_ = 630/60 nm). The best variant identified in the 161X/163X library, named as LSSmCherry0.1, possessed the Ile161Ser and Gln163Glu mutations and exhibited a blue-shifted excitation at 450 nm (Fig 3A), presumably due to establishment of an ESPT pathway that involved the introduced glutamate. However, further spectroscopic characterization of LSSmCherry0.1 revealed that a substantial portion of the protein formed a green fluorescent chromophore, and a substantial amount of short Stokes shift red fluorescence co-existed with the long Stokes shift fluorescence. In the attempt to further improve mCherry2-I161S/I163E by elimination of the undesirable green and short Stokes shift red fluorescence, saturation mutagenesis was performed at position 143, which is in the close vicinity of the chromophore. The variant with the highest ratio of long Stokes shift fluorescence relative to short Stokes shift and green fluorescence was mCherry2-W143L/I161S/Q163E, which was designated as LSSmCherry0.2. LSSmCherry0.2 exhibits exclusively long Stokes shift red fluorescence with no noticeable short Stokes shift red fluorescence (Fig 3B). This mutant was subjected to two rounds of random mutagenesis using error-prone polymerase chain reaction (EP-PCR) and screened for further improved long Stokes shift fluorescence brightness. The final variant, LSSmCherry1 (equivalent to mCherry2-A6T/W143L/I161S/Q163E/A217S), has a 160 nm Stokes shift, with an excitation maximum at 450 nm and an emission maximum at 610 nm (Fig 1C and 1D). The molar extinction coefficient (ε) and quantum yield (Φ) of LSSmCherry1 was determined to be 35,000 M^−1^cm^−1^ and 0.29, respectively (Table 1). Mutated residues 143, 161, 163 and 217 all have their side chains directed inside the β-barrel structure of the fluorescent protein and are in close proximity to the chromophore (Fig 1C). The Ala6Thr mutation is located far from the chromophore at the N-terminus of the protein, and may be facilitating protein folding. To assess the cytotoxicity of LSSmCherry1, it was subjected to the bacterial cytotoxicity assay (Fig 2F). This assay revealed that LSSmCherry1 has cytotoxicity that is similar to that of mCherry.

### Directed evolution of a red-shifted mCherry variant

During screening of the mCherry2 161X/163X library it was noticed that a number of colonies appeared blue or purple to visual inspection. A blue or purple visible color is typically associated with far-red shifted RFPs such as mNeptune [28]. The brightest of these colonies was picked and cultured for further analysis. Gene sequencing revealed this variant to be mCherry2-I161G/Q163G, and spectral analysis confirmed that it had a substantially red shifted fluorescence excitation and emission maxima at 598 nm and 625 nm, respectively. This spectral profile is similar to that of the previously reported far-red RFP mRaspberry [26]. mCherry2-I161G/Q163G (designated as RDSmCherry0.1; Table 1) was subjected to further engineering aimed at improving the brightness and shifting the excitation and emission further to the red.

One strategy for red-shifting the fluorescence of a fluorescent protein is the introduction of a π—π stacking interaction through mutation of an appropriately oriented residue near the chromophore to tyrosine. Such a strategy was employed to engineer a yellow fluorescent protein from avGFP [40] (Thr203Tyr mutation) and the red shifted mGrape variant from DsRed-derived mRFP1.1 (Ile197Tyr mutation) [28]. In an attempt to introduce an analogous π—π interaction into RDSmCherry0.1, positions 197 and 195 were randomized by saturation mutagenesis. Screening for the most red-shifted mutant in the 195X/197X library led to the identification of mCherry2-I161G/Q163G/V195C/I197Y, which was designated RDSmCherry0.2 (Fig 3C; Table 1). Despite the successful introduction of Tyr at position 197, this variant was only modestly red-shifted relative to RDSmCherry0.1, with an excitation maximum at 600 nm and an emission maximum at 630 nm. Multiple additional rounds of saturation mutagenesis and EP-PCR produced RDSmCherry0.5 with excitation maximum further red-shifted to 604 nm and emission maximum shifted to 636 nm (Table 1). Compared with the original template mCherry2, RDSmCherry0.5 had 7 mutations: Val16Ser, Ala44Cys, Ala145Pro, Ile161Gly, Gln163Gly, Val195Cys, and Ile197Tyr. With the exception of Ala145Pro, all of these residues have their side chains inside the β-barrel and directed towards the chromophore.

During spectral characterization of RDSmCherry0.5, it was found that this variant exhibited a distinctive blue fluorescence with excitation at 400 nm and emission at 462 nm. This blue fluorescent species is most likely a mTagBFP-like chromophore species [41,42], which is a putative intermediate in the chromophore formation mechanism [43]. Further analysis of earlier versions showed that RDSmCherry0.2 had a similar blue fluorescence of excitation at 400 nm and emission at 466 nm (Fig 3D), while RDSmCherry0.1 did not exhibit any blue fluorescence. This led us to speculate that the mutations at position 195 and 197 were responsible for the undesirable blue fluorescence. Accordingly, another round of saturation mutagenesis on 195 and 197 was performed on the template of RDSmCherry0.5 with screening for maximal far-red fluorescence and minimal blue fluorescence. Thorough screening of this library led to the identification of mCherry2-V16S/A44C/A145P/I161G/Q163G/V195C, which was designated as RDSmCherry1 (Fig 1E and 1F). Reversion of Tyr197 to Ile in the final round of engineering was associated with the complete disappearance of the blue fluorescence and an increase in red intensity. This increase in red intensity is presumably due to a decreased fraction of chromophores accumulating in the blue intermediate state (or being diverted to a similarly fluorescent dead-end product). RDSmCherry1 (ε = 55,400 M^−1^cm^−1^ and Φ = 0.09) is slightly blue shifted relative to RDSmCherry0.5 with an excitation maximum at 600 nm and a peak emission at 630 nm (Table 1). The bacterial cytotoxicity assay demonstrated that RDSmCherry1 has cytotoxicity similar to that of mCherry and LSSmCherry1 (Fig 2G), indicating that the decreased cytotoxicity of mCherry2 was lost during directed evolution.

## Discussion

Inspired by the observation of decreased bacterial cytotoxicity for mCherry2 (relative to mCherry), we have used protein design and directed evolution to develop new RFP variants with low cytotoxicity to *E*. *coli* and altered fluorescence excitation and emission profiles. However, at this stage of development, none of these variants are improved relative to the current state of the art monomeric RFPs in their respective classes. That is, FusionRed is a monomeric RFP with low cytotoxicity, excellent performance in fusions expressed in mammalian cells, and brightness that is practically identical to mCherry2 [18]. mCardinal, which is derived from *Entacmaea quadricolor* eqFP578, is 3.3× brighter than RDSmCherry1 and has an emission peak that is 29 nm more red-shifted [30]. hmKeima8.5 has similar brightness to LSSmCherry1 and has been demonstrated to provide particularly high brightness and photostability when expressed in mammalian cells [44]. The recently reported CyOFP1 is 3x brighter than LSSmCherry1 [45]. So, while these new mCherry variants are unlikely to find immediate use in live cell imaging applications, they do provide insight into the influence of the protein structure on mCherry’s fluorescence and cytotoxicity properties and may serve as starting points for future probe development efforts.

It has been suggested that fluorescent protein aggregation may lead to cytotoxicity [36], but this mechanism does not explain why some highly monomeric fluorescent proteins, such as mCherry, can be quite cytotoxic. Other potential sources of cytotoxicity could include the generation of illumination-dependent generation of reactive oxygen species (i.e., phototoxicity) and the maturation-dependent generation of hydrogen peroxide [46]. While different fluorescent protein variants do exhibit different degrees of phototoxicity [47], this mechanism cannot explain the cytotoxicity differences described here and elsewhere, because cells are grown in the dark. Variants that fold faster and more efficiently may produce higher levels of hydrogen peroxide than variants that fold slower and less efficiently. However, if this were the primary mechanism of cytotoxicity, the degree of cytotoxicity would be expected to correlate with the observed brightness of a cell. As the example of mCherry versus the slightly brighter and less cytotoxic mCherry2 demonstrates, this relationship does not hold.

While this work cannot provide a definitive answer as to the mechanism of mCherry’s cytotoxicity, examination of the mutations that separate mCherry and mCherry2 does hint at a possible explanation. Notably, mCherry2 has three fewer surface-exposed lysines than mCherry. Two of these lysines were mutated to non-charged residues and one was mutated to an arginine that would retain a positive charge. Yet another residue was mutated from a non-charged asparagine to a negatively charged aspartate. Overall, these substitutions result in a net change in charge of negative three for the whole protein and a corresponding substantial drop in isoelectric point. Intriguingly, these 4 substitutions are retained in the mCherry1.5 variant that exhibits even less cytotoxicity than EGFP. This result tentatively suggests that a primary mechanism of cytotoxicity in *E*. *coli* may be disruptive interactions with negatively charged biomolecules: anionic phospholipids, DNA, and RNA being the three most obvious candidates. Such a mechanism could explain why fluorescent protein cytotoxicity in bacteria and mammalian cells appears to be correlated [36].

Due to its decreased cytotoxicity, mCherry2 is a promising template for the development of a palette of new variants with altered excitation and emission wavelengths. mCherry and other DsRed-derivatives have been evolved with many different screening pressures, including blue-shifted emission [9], red shifted emission [26], and increased photostability [10], among others. In this work we initially identified red-shifted and long Stokes shift variants in a library in which positions Ile161 and Gln163 of mCherry2 were randomized to all 20 amino acids, and then subjected these prototypes to multiple rounds of directed evolution. These residues were chosen due to their close proximity to the phenolate group of the chromophore and the fact that mutations at these positions are often critical to modifying the properties of DsRed-derivatives (e.g., as in the mFruits [9] and photoswitchable variants [48]). Indeed, introduction of a glutamate or aspartate at positions 161 and 163 (structurally aligned with positions 158 and 160 of mKate) is a robust approach for introducing a long Stokes shift phenotype in various RFPs [25]. For example, Met160Glu is the key substitution to convert mKate into LSSmKate1 [23]. Screening of the 161/163 library of mCherry led to the rediscovery of the previously reported Gln163Glu mutation. Further directed evolution allowed us to effectively eliminate the substantial green and short Stokes shift red fluorescence of LSSmCherry0.1 and ultimately produce the variant we have designated LSSmCherry1. LSSmCherry1 exhibits essentially no short Stokes shift fluorescence and has brightness that is 4.0× and 2.3× that of LSSmKate1 and LSSmKate2 [23], respectively, and 0.9× that of hmKeima8.5 [44].

The same 161/163 library that gave rise to LSSmCherry0.1 also gave rise to red shifted RDSmCherry0.1 (excitation red-shifted by 11 nm and emission red-shifted by 15 nm), which was identified due to the blue appearance of a colony. Interestingly, the key mutations, Ile161Gly and Gln163Gly, must leave a solvent-filled cavity adjacent to the chromophore. Further directed evolution ultimately led to RDSmCherry1 that is less red-shifted and dimmer than a substantial number of previously reported mKate derivatives [30]. The development of RDSmCherry is very similar to that of mRouge in terms of the choice of starting template, screening criteria, critical mutation positions and the final variant spectroscopic properties [29]. A key difference between these two studies is that the development of mRouge was aided by computational library design, while here we relied solely on colony-based screening of libraries generated by EP-PCR and targeted codon randomization. These different approaches led to the selection of distinctly different sets of mutations with the most notable difference being the combination of Ile161Gly and Gln163Gly in RDSmCherry1 compared to Ile161Met and Gln163Met in mRouge. Overall, the convergent evolution of mRouge and RDSmCherry1 to produce relatively dim variants provides further support for the notion that DsRed-derived RFPs are not readily amenable to evolution for extreme red shift. In contrast, eqFP578 from *Entacmaea quadricolor* has been the progenitor of monomeric RFPs with quantum yield as high as high as 0.28 [30] and fluorescence emission peaks as red-shifted as 670 nm [49]. We suggest that future efforts to develop red-shifted RFPs should focus on epFP578 derivatives or other non-*Discosoma* sp. RFP templates.

## Materials and methods

### General methods and materials

All synthetic DNA oligonucleotides for cloning and library construction were purchased from Integrated DNA Technologies. Taq DNA polymerase (New England Biolabs) was used for EP-PCR. PCR products and products of restriction digests were purified using GeneJET gel extraction kit (Thermo Scientific) according to the manufacturer’s protocols. Restriction enzymes and ligases were purchased from New England Biolabs or Thermo Scientific. The cDNA sequences were confirmed by dye terminator cycle sequencing using the BigDye Terminator v3.1 Cycle Sequencing Kit (Applied Biosystems). Sequencing reactions were analyzed at the University of Alberta Molecular Biology Service Unit.

### Bacterial growth rate comparison

Electrocompetent *E*. *coli* was transformed with RFP or EGFP encoding plasmid with the same pBAD/His-B vector. The transformed bacteria were then cultured separately in two tubes of LB medium with 0.1 mg/ml ampicillin overnight. 50 μL of *E*. *coli* cultures from each tubes was mixed and cultured in a tube with 4 mL fresh LB medium supplemented with 0.1 mg/ml ampicillin and 0.02% (w/v) L-arabinose for 14 to 16 hours. Inoculation of 50 μL from the mixed culture to fresh medium was repeated every day for 4 days. Each overnight culture was plated on agar plates supplemented with 0.4 mg/ml ampicillin and 0.02% (w/v) L-arabinose. All the plates were imaged using a custom built colony imaging system under green (470/40 nm excitation and 510/20 nm emission) and red (560/40 nm excitation and 630/60 nm emission) channel respectively. Percentages of EGFP and RFP-expressing bacteria were calculated based on the fluorescent colony counts obtained from the images.

### Site directed saturation mutagenesis and random mutagenesis

Directed evolution of LSSmCherry1 and RDSmCherry1 was carried out by site directed saturation mutagenesis and EP-PCR using plasmids encoding mCherry2 as template. All site-directed mutagenesis was performed using the Quikchange lightning mutagenesis kit (Agilent) and primers designed according to the manufacturers guidelines with the degenerate codon (NNK) for the intended mutation positions. EP-PCR was performed using unbalanced dNTP concentrations and increased concentration of MgCl_2_ (50 mM), and the introduction of MnCl_2_ (10 mM) to further decrease the fidelity of Taq DNA polymerase. Products were digested with XhoI and HindIII and ligated into pBAD/His B vector (Life technologies) digested with the same two enzymes, and used to transform the electrocompetent *E*. *coli* strain DH10B ElectroMax (Life technologies) using a Micropulser electroporator (Bio-Rad), which were then plated on agar plates containing LB medium supplemented with 0.4 mg/ml ampicillin and 0.02% (w/v) L-arabinose. Plates were incubated for overnight at 37°C prior to screening.

### Library screening

*E*. *coli* colonies expressing the mutation libraries were grown on 10 cm Petri dishes. In order to screen libraries for variants that exhibited brighter long Stokes shift red fluorescence, a screening method was developed for long Stokes shift RFPs when expressed in colonies of *E*. *coli*. The custom built fluorescent colony imaging system equipped with filter sets (Chroma) for 470/40 nm excitation with 630/60 nm emission (i.e., for long Stokes shift fluorescence) and 560/40 nm excitation with 630/60 nm emission (i.e., for red fluorescence) is used to acquire both long Stokes shift and red fluorescence images of the Petri dish. Colonies exhibiting the highest intensity ratio (long Stokes shift fluorescence intensity / red fluorescence intensity) between these channels were picked and cultured for further spectral confirmation. For far-red RFP screening, colony images were acquired with filter set 622/36 nm excitation and 680/40 nm emission. Colonies exhibiting the highest intensities were picked and cultured for further spectral confirmation. Single colonies were picked, inoculated into 4 ml of LB medium with 0.1 mg/ml ampicillin and 0.02% (w/v) L-arabinose and then cultured overnight. Protein was extracted using B-PER bacterial protein extraction reagent (Thermo Scientific) as per manufacturer guidelines. Screening of the extracted protein was performed with a Safire2 fluorescence plate reader (Tecan) by measuring protein fluorescence. The brightest long Stokes shift variant or most red-shifted variant was selected to be the template for the following round of library creation.

### Protein purification and in vitro characterization

To purify the RFPs, electrocompetent *E*. *coli* strain DH10B Electromax (Life Technologies) was transformed with the plasmid of interest using Micropulser electroporator (Bio-Rad). Transformed bacteria were cultured overnight on agar plates containing LB and 0.4 mg/ml ampicillin. A single colony was picked and grown overnight in 4 mL LB supplemented with 0.1 mg/ml ampicillin at 37°C. The 4 mL culture was then used to inoculate 250 mL of LB medium with ampicillin and grown to an optical density of 0.6. Protein expression was induced with the addition of 0.02% arabinose and the culture was grown overnight at 37°C. Bacteria were harvested at 10,000 rpm, 4°C for 10 min, lysed using a cell disruptor (Constant Systems) and then clarified at 14,000 rpm for 30 min. Protein was purified from the supernatant by Ni-NTA affinity chromatography (ABT) according to the manufacturer’s instructions. The buffer of the purified protein was exchanged with 10 mM Tris-Cl, 150 mM NaCl, pH 7.3 with Amicon ultracentrifugal filter (MWCO 10,000 Da). Molar extinction coefficients (ε) were measured by the alkali denaturation method. Briefly, the protein was diluted into Tris buffer or 1 M NaOH and the absorbance spectra recorded under both conditions. The value of ε was calculated assuming the denatured RFP chromophore has an ε = 44,000 M^-1^cm^-1^ at 452 nm. Fluorescence quantum yields (Φ) were determined using LSSmKate2 or mCherry as standards. Fluorescence intensity as a function of pH was determined by dispensing 2 μL of the protein solution into 50 μL of the desired pH buffer in triplicate into a 396-well clear-bottomed plate (Nunc) and measured in a Safire2 plate reader.

### Live cell imaging

To construct Orai1-mCherry, Orai1-mCherry1.5, Orai1-mEGFP, CytERM-mCherry, and CytERM-mCherry1.5, the genes for mCherry, mCherry1.5, and mEGFP were first amplified with a 5’ primer with an *AgeI* site and a 3’ primer with a *NotI* site. The purified PCR products were then digested and ligated into a similarly digested pOrai1-YFP or pCytERM-mEGFP. The plasmid encoding Orai-YFP was acquired from Addgene, plasmid number 19756. The plasmid encoding CytERM-mEGFP was gifted from Professor Erik Snapp at Albert Einstein College of Medicine. HeLa cells and HEK cells were maintained in Dulbecco’s Modified Eagle Medium (DMEM) supplemented with 10% fetal bovine serum (FBS, Sigma) and Glutamax (Life Technologies) and incubated at 37°C with 5% CO_2_. Transient transfections were performed using Turbofect (Thermo Scientific) according to the manufacturer’s guidelines. Transfected cells were imaged using either an Axiovert 200M (Zeiss) or a Nikon Eclipse Ti. The Axiovert 200M (Zeiss) was equipped with a 75 W xenon-arc lamp, a 40× objective lens (NA = 1.3, oil), a 14-bit CoolSnap HQ2 cooled CCD camera (Photometrics), and driven by open source Micro-Manager software. The Nikon Eclipse Ti microscope was equipped with a 150 W Lumen 200 metal halide lamp (Prior Scientific), a 16-bit 512SC QuantEM CCD (Photometrics), a 25% neutral density filter, a 40× objective (NA = 0.95, air), and driven by a NIS-Elements AR 3.0 software package (Nikon).

## Supporting information

S1 Table Amino acid substitutions in new RFPs described in this work. (PDF)

S1 Fig Representative fluorescence images of cells expressing mCherry and mEGFP fusions. (PDF)

S1 Dataset Numerical data for all figures. (XLSX)
