# Supplementary material for: Engineering of mCherry variants with long Stokes shift, red-shifted fluorescence, and low cytotoxicity
Source: PLoS One. 2017 Feb 27;12(2):e0171257. doi: 10.1371/journal.pone.0171257 (PMC5328254; doi:10.1371/journal.pone.0171257)
Supplement: S1 Table — (PDF) [file pone.0171257.s001.pdf]

S1 Table. Amino acid substitutions in new RFPs described in this work.

Protein name	Additional mutations
mCherry2	mCherry + K92N + K138C + K139R + S147T + N196D + T202L
mCherry1.5	mCherry + K92N + K138C + K139R + N196D
LSSmCherry0.1	mCherry2 + I161S + Q163E
LSSmCherry0.2	LSSmCherry0.1 + W143L
LSSmCherry1	LSSmCherry0.2 + A6T + A217S
RDSmCherry0.1	mCherry2 + I161G + Q163G
RDSmCherry0.2	RDSmCherry0.1 + V195C/I197Y
RDSmCherry0.5	RDSmCherry0.2 + V16S + A44C + A145P
RDSmCherry1	RDSmCherry0.5 + Y197I
