# Supplementary material for: Engineering of mCherry variants with long Stokes shift, red-shifted fluorescence, and low cytotoxicity
Source: PLoS One. 2017 Feb 27;12(2):e0171257. doi: 10.1371/journal.pone.0171257 (PMC5328254; doi:10.1371/journal.pone.0171257)
Supplement: S1 Fig — (PDF) [file pone.0171257.s002.pdf]

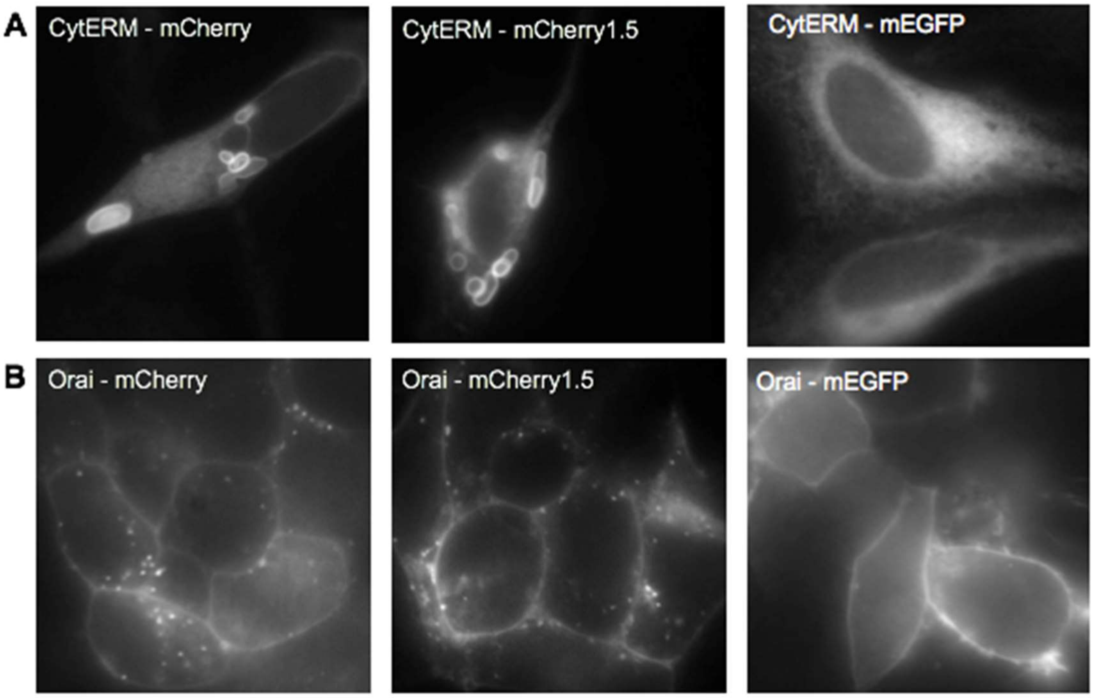

S1 Fig. Representative fluorescence images of cells expressing mCherry and mEGFP fusions. CytERM is an endoplasmic reticulum (ER) signal-peptide which anchors its downstream protein to the cytoplasmic side of ER. Orai1 is a calcium selective ion channel named as calcium release-activated calcium channel protein 1. These two fusion partners were chosen because they were previously reported as “challenging” targets for mCherry [38,51]. For CytERM-mCherry cells, we observed some cells with correct localization and no OSER structures, but at a substantially lower percentage than the 80% to 95% previously reported [52,53]. This discrepancy could be due to differences in expression level which could in turn be affected by numerous experimental variables including cell confluence, amount of plasmid DNA used for transfection, transfection time period, and the time point for observation.

Supplementary References

51. Costantini LM, Fossati M, Francolini M, Snapp EL. Assessing the tendency of fluorescent proteins to oligomerize under physiologic conditions. Traffic. 2012;13: 643-649.

52. Cranfill PJ, Sell BR, Baird MA, Allen JR, Lavagnino Z, de Gruiter HM, et al. Quantitative assessment of fluorescent proteins. Nat Methods. 2016;13: 557-562.

53. Bindels DS, Haarbosch L, van Weeren L, Postma M, Wiese KE, Mastop M, et al. mScarlet: a bright monomeric red fluorescent protein for cellular imaging. Nat Methods. 2017;14: 53-56.
